# Low-loss silicon core fibre platform for mid-infrared nonlinear photonics

**DOI:** 10.1038/s41377-019-0217-z

**Published:** 2019-11-21

**Authors:** Haonan Ren, Li Shen, Antoine F. J. Runge, Thomas W. Hawkins, John Ballato, Ursula Gibson, Anna C. Peacock

**Affiliations:** 1 Optoelectronics Research Centre, University of Southampton, Southampton, SO17 1BJ UK; 2 Wuhan National Laboratory for Optoelectronics (WNLO), Huazhong University of Science and Technology, 430074 Wuhan, China; 3 Department of Materials Science and Engineering, Clemson University, Clemson, SC 29634 USA; 4 Department of Physics, Norwegian University of Science and Technology (NTNU), N-7491 Trondheim, Norway; 5 Department of Applied Physics, KTH Royal Institute of Technology, 10044 Stockholm, Sweden; 6 Present Address: The Institute of Photonics and Optical Science (IPOS), School of Physics, University of Sydney, Sydney, NSW 2006 Australia

**Keywords:** Nonlinear optics, Silicon photonics, Mid-infrared photonics

## Abstract

Broadband mid-infrared light sources are highly desired for wide-ranging applications that span free-space communications to spectroscopy. In recent years, silicon has attracted great interest as a platform for nonlinear optical wavelength conversion in this region, owing to its low losses (linear and nonlinear) and high stability. However, most research in this area has made use of small core waveguides fabricated from silicon-on-insulator platforms, which suffer from high absorption losses of the use of silica cladding, limiting their ability to generate light beyond 3 µm. Here, we design and demonstrate a compact silicon core, silica-clad waveguide platform that has low losses across the entire silicon transparency window. The waveguides are fabricated from a silicon core fibre that is tapered to engineer mode properties to ensure efficient nonlinear propagation in the core with minimal interaction of the mid-infrared light with the cladding. These waveguides exhibit many of the benefits of fibre platforms, such as a high coupling efficiency and power handling capability, allowing for the generation of mid-infrared supercontinuum spectra with high brightness and coherence spanning almost two octaves (1.6–5.3 µm).

## Mid-infrared photonics: silicon core fibres

Tapered optical fibres with a silicon core can act as a high-brightness supercontinuum source in the mid-infrared. The silicon core fibre (SCF) was designed and fabricated by an international team of researchers from UK, China, Australia, USA and Norway. The fibre was fabricated by placing a silicon rod inside a silica glass capillary which is coated with calcium oxide and the composite structure is then heated and drawn into a fibre. Following drawing, the fibre was tapered so that core size decreased in size from 10 to 2 µm. When pumped with femtosecond pulses from an optical parametric oscillator at a wavelength of around 3 µm the tapered SCF emits broadband coherent light spanning two octaves (1.6–5.3 µm). Potential applications for such a source include mid-IR gas sensing, spectroscopy and free-space communications.

## Introduction

The mid-infrared (mid-IR) region is an important spectral region in which strong molecular absorption bands and atmospheric transmission windows can be exploited for practical use in medicine, food production, imaging, environmental monitoring, and security^1,2^. For applications that require broad spectral bandwidths, such as spectroscopic sensing^3^ and high-resolution imaging^4^, supercontinuum (SC) sources based on extreme nonlinear phenomena have emerged as the most popular option. To be effective, these sources must exhibit several key features, including coherence, high brightness, robustness, stability, and for healthcare applications, safe handling^5^. Mid-IR SC spectra have been demonstrated in a range of material systems, in both fibre and planar platforms. To date, the broadest and brightest spectra have been demonstrated in fibre systems made from non-silica soft glasses (e.g., chalcogenides^6^, fluorides^7^, or tellurites^8^), primarily due to their capability to handle high power levels. However, there are challenges when working with these materials, as they are not as stable or robust as their silica counterparts and, in the case of the chalcogenides, they often contain toxic compounds. Alternatively, planar-based SC systems employing highly nonlinear group IV materials (e.g., silicon) and compound III–V semiconductors (e.g., GaAs and AlGaAs) can avoid these issues and offer advantages in terms of compactness and on-chip integration^9,10^, which are important considerations for the development of portable systems. In this case, the trade-off is that these small core waveguides suffer from low power conversion efficiency due to both the high on-chip coupling losses (typically 5–10 dB per facet) and propagation losses associated with increased core/cladding interactions^11^. As a result, the best demonstrations of SC generation in silicon-on-insulator (SOI) waveguides, which are the most common semiconductor waveguide platforms, have so far been limited to wavelengths <3.3 μm^12,13^. With a view towards extending the long wavelength edge, more complicated structures (i.e., suspended waveguides^14^) and material systems (e.g., silicon-on-sapphire^15-17^, silicon–germanium (SiGe)-on-silicon^18^, and suspended III–V semiconductors-on-silicon waveguides^19^) have been considered, though these come with increased fabrication costs and integration complexity.

Silicon core fibres (SCFs) represent an emerging platform that combines the benefits of fibre geometry with the advantages of semiconductor material systems. As these fibres are clad in silica, they are robust, stable, and fully compatible with standard fibre fabrication procedures, thus increasing the device yield and reducing costs^20^. Until recently, one of the main limitations to producing SCFs using a fibre-drawing tower was obtaining the small, few-micrometre core sizes needed to achieve efficient nonlinear processing. This is because, at the temperatures required to soften the silica cladding, the silicon core is molten, and high drawing speeds can lead to break-up of the core due to Rayleigh instabilities^21^. To overcome this hurdle, a post-draw tapering procedure was developed, which allows for more accurate control over the flow of the molten core, resulting in crystalline silicon optical fibres with the smallest core and with losses comparable to their planar waveguide counterparts^22^. This achievement has allowed for the first demonstration of nonlinear propagation in the drawn SCF platform, extending from the telecom band up to the mid-IR region^23^. Similar to planar waveguides, SCFs have shown great potential for nonlinear processing in the mid-IR due to the reduced effects of two-photon absorption (TPA), but with a number of interesting advantages. First, the larger SCF core sizes reduce the interaction with the silica cladding and increase the threshold for higher-order absorption mechanisms, allowing for higher power operation compared with the small core planar structures. Second, fibre-tapering procedures can be used to precisely engineer the dispersion profile to improve the phase matching of nonlinear processes and enhance SC generation^24^. Finally, the SCFs exhibit key fibre features, such as polarisation independence and high power-handling capabilities, and can be directly spliced to other fibre components^25^, including the increasingly popular mid-IR fibre lasers^26,27^, which opens a route to more efficient and elegantly packaged mid-IR systems.

In this work, we demonstrate a compact SCF platform capable of achieving a high-brightness SC spectrum spanning almost two octaves, from the near infrared into the mid-IR. The fibre was carefully designed in the shape of an asymmetric taper, which allows for improved coupling to achieve efficient nonlinear propagation, while minimising the interaction of the long wavelength light with the silica cladding at the output. A femtosecond optical parametric oscillator (OPO) was used to pump the SCF at wavelengths of ~3 μm near the zero-dispersion wavelength (ZDW) of the tapered waist to generate a spectrally bright continuum covering 1.6–5.3 μm (∼3700 nm) with high coherence. The spectral broadening observed in the SCF represents the largest bandwidth generated in a silicon core/silica-clad waveguide to date, with the long wavelength edge being pushed well beyond the silica absorption edge. The low propagation loss of our tapered SCF (<1 dB cm^−1^ in the mid-IR region) enables a power conversion efficiency of ∼60%, which is higher than that in previous demonstrations of group IV-based waveguides^12–18^ and is comparable to non-silica fibre-based SC systems^28^. Furthermore, numerical simulations are used to show that the SCF platform can support SC generation extending out to 8 μm, covering the entire transparency window of the silicon core^29^. This work provides a crucial step towards developing robust and practical all-fibre mid-IR broadband sources for next-generation healthcare and communication systems.

## Results

### Design of a low-loss mid-IR nonlinear SCF platform

**Fig. 1:**
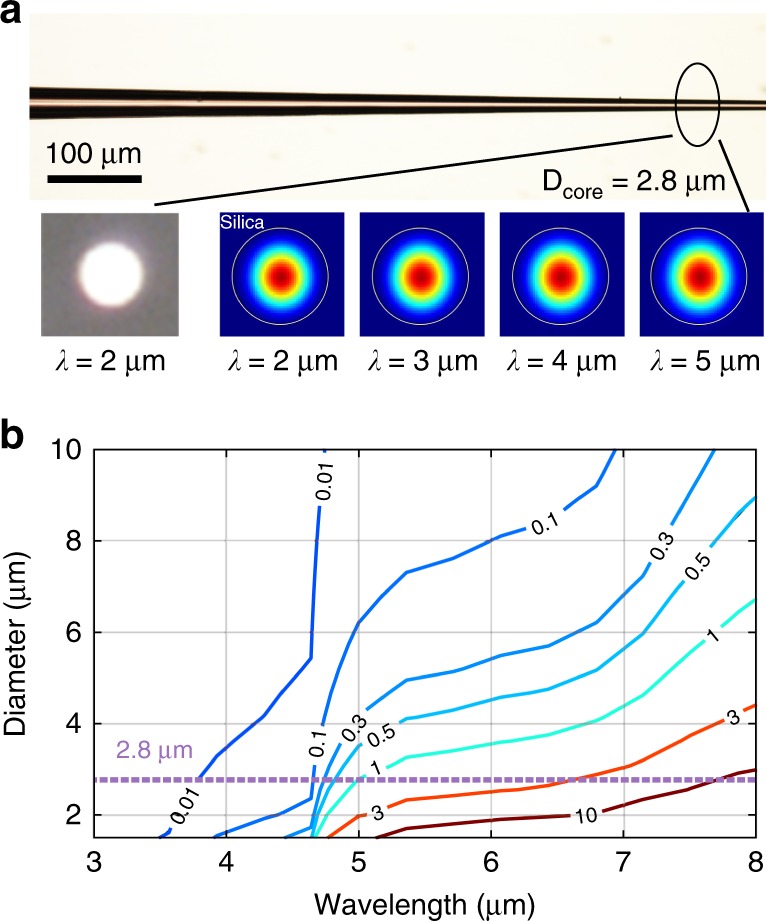
**a** Microscopy image of a tapered SCF with varying core diameters. Inset: measured output mode image (2 µm scale bar) and simulated mode profiles of a 2.8 µm core SCF at different wavelengths, as labelled. White lines highlight the boundary between the silicon core and silica cladding. **b** Contour map showing simulated absorption losses for the SCFs in dB mm^−1^ as functions of wavelength and fibre core diameter.

The SCFs used in this work were fabricated using the molten core method (MCM) followed by tapering (as described in ref. ^30^); both procedures were adapted from conventional silica fibre processing (see the “Methods” section). Significantly, as well as decreasing the fibre dimensions, the post-draw tapering method also serves to improve the crystallinity of the silicon core to almost single-crystal-like quality, thus reducing the transmission losses^31^. Figure 1a shows an optical microscopy image of a tapered SCF with a constantly decreasing core diameter from 10 to 2 μm. As bulk silicon is transparent up to 8 μm, the linear propagation losses observed in silicon core/silica clad waveguides at longer mid-IR wavelengths primarily originate from the overlap of the optical mode with the silica clad glass, which absorbs at these wavelengths. Thus, to achieve low propagation losses (<1 dB cm^−1^) in this region, the cross-sectional geometry of the SCF is tailored to minimise this interaction for all generated wavelengths. As an illustration, Fig. 1b shows the linear losses at wavelengths beyond 3 μm for the SCFs with different diameters, as calculated via finite-element method (FEM) simulations of the modal properties. In these simulations, the real and imaginary parts of the complex refractive indices of silicon and silica are adapted from the literature^32,33^. For example, an SCF with a core diameter of 2.8 μm exhibits low propagation losses (<1 dB cm^−1^) for wavelengths up to ∼4.7 μm and only a modest increase beyond this. This result is expected from the images of the fundamental mode for a fibre of this core size, shown in the inset of Fig. 1a, which confirms that the mode is well confined within the core over this wavelength range. Moreover, considering that typical lengths for silicon-based nonlinear devices are on the order of millimetres, the optical transmission in micron-sized SCFs remains tolerable for even longer wavelengths, especially when compared with the submicron dimensions used for on-chip waveguides^11^. For example, an optical beam at a wavelength of 6 μm propagating through the length of a 1 mm SCF with a 2.8 μm core diameter experiences only an extra 2 dB loss.

**Fig. 2:**
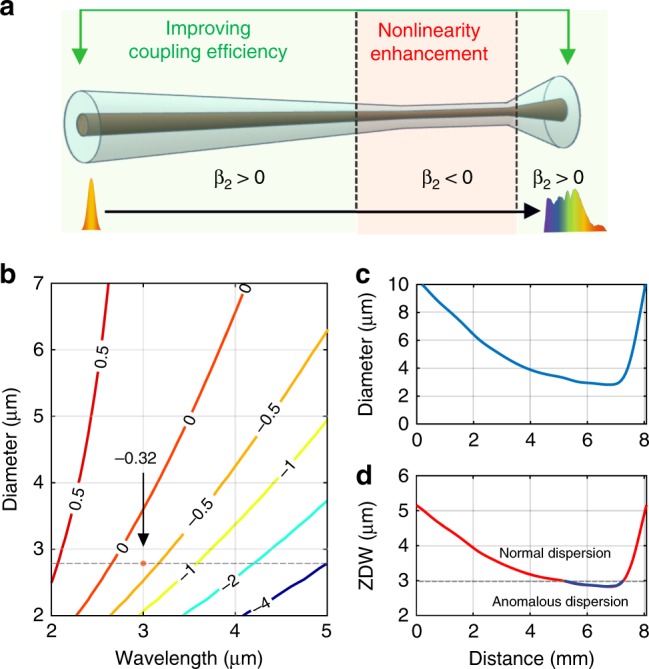
**a** Schematic of the asymmetric taper design. **b** Contour map of the GVD parameter *β*_2_ (ps^2^ m^−1^) as a function of the core diameter and wavelength of the SCFs. **c** Profile of the core diameter variation in the fabricated tapered fibre. **d** ZDW profile of the fabricated tapered SCF.

The design of the tapered SCF used in our work is illustrated schematically in Fig. 2a. The taper profile was optimised using numerical modelling of the nonlinear pulse propagation, as described in Supplementary Information Section I. Obtaining the broadest spectrum requires a careful balance of the tapered dimensions to maximise the nonlinear processes while minimising the losses of the long wavelength light. This led us to design an asymmetric profile in which a large input core is slowly tapered down to ensure maximum coupling into the waist, reducing mode coupling and radiation loss, while a sharp up-taper is employed at the output to reduce the interaction with the lossy cladding. To achieve broadband mid-IR SC generation, the SCF was optimised to be pumped at a wavelength of ~3 µm. This pump wavelength was chosen because the nonlinear refractive index of the silicon core is still relatively large, while the multiphoton absorption is modest as the three-photon absorption (3PA) edge is approached^34^. Figure 2b shows a contour map of the group velocity dispersion (GVD) parameter, *β*_2_, as calculated via the well-known eigenvalue equation^35^, for the SCFs as functions of both wavelength and fibre core diameter. For the pump to access the anomalous dispersion region required for efficient SC generation^36^, the SCF only needs to be tapered down to a core diameter of <3.5 µm, which helps to ensure low propagation losses for wavelengths up to 6 µm, as illustrated in Fig. 1b. We note that although these micron-sized fibres support multiple guided modes, with careful coupling into the core, it is possible to propagate most of the light in the fundamental mode (see Supplementary Information Section III)^37^. The exact profile of a fabricated tapered SCF is plotted in Fig. 2c. The fibre is gradually tapered down from a 10 μm core over the first 5.5 mm to a 2.8 µm diameter waist of 1.5 mm in length, followed by a 1 mm long inverse taper back up to a 10 μm core at the output. The waist length was chosen so that it was sufficiently long to induce a significant nonlinear phase shift but without introducing prohibitively high losses for longer wavelengths. The corresponding map of the ZDW for this taper is shown in Fig. 2d, indicating that the waist region is in the anomalous dispersion regime for the 3 μm pump. Interestingly, the varying dispersion profile of the taper provides another benefit in that it allows for multiple phase-matching conditions to be satisfied simultaneously, which can result in a flatter and more coherent SC^38,39^. Although varying dispersion profiles can also be produced by splicing different sections of fibre together, each with a different dispersion parameter^40^, tapering is preferred for our SCFs owing to their short lengths and high core/cladding index contrast, which can result in high losses at the coupling interfaces if the mode properties are not well matched. By monitoring the low power transmission through the tapered SCFs, the average linear propagation losses are extracted to be within the range of 0.2–3 dB cm^−1^ over the wavelength region of 1.7–3.7 µm (see Supplementary Information Section II), which are comparable to the lowest losses reported in on-chip silicon waveguides with similar dimensions^16,41^.

### Experimental setup for SC measurements

**Fig. 3:**
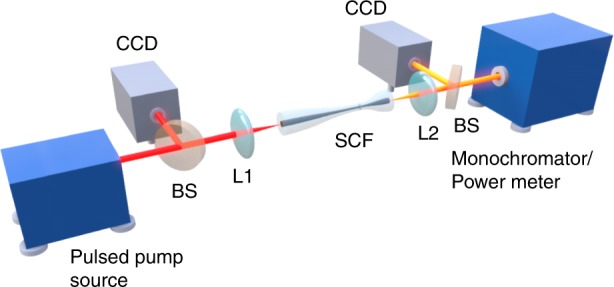
Experimental setup of SC generation in a tapered SCF. The pulsed pump source is coupled to the tapered SCF with a lens (L1). The output light is collected with a second lens (L2) and measured by a power meter or a monochromator via free space alignment. Beam splitters (BSs) are used to direct light at the fibre input and output facets onto CCD cameras to monitor the coupling.

The experimental setup for SC generation is shown in Fig. 3. A Ti:sapphire-pumped OPO with an ∼100 fs (FWHM) pulse duration and an 80 MHz repetition rate was used as the pulse source. However, this source can easily be replaced with a femtosecond fibre laser that operates around the 3 µm regime^42^, to enable a compact and robust all-fibre SC source, similar to that proposed using a chalcogenide-based fibre taper in ref. ^38^. The OPO was tuned to 3 µm and launched into the SCF via a black diamond objective lens L1 with a numerical aperture (NA) of 0.56 to achieve a waist radius at the input facet of 2.9 µm, which was chosen to best match the fundamental mode radius (see Supplementary Information Section III). The output light was collected by another black diamond objective lens L2 (NA = 0.85). The coupling loss at the input fibre facet is estimated to be only 1.4 dB (excluding Fresnel reflections) at this pump wavelength. It is worth noting that this improved coupling efficiency, due to the taper structure, shows at least a 3 dB improvement than previous demonstrations for on-chip mid-IR silicon waveguides^11–18^, which we attribute to better mode matching between the pump laser and the circularly symmetric fibre. CCD mid-IR cameras were employed at the input and output facets to facilitate coupling to the fundamental mode, as seen in the inset of Fig. 1a. Finally, the output spectra were recorded on a monochromator (Bentham TMc300).

### Spectral evolution in tapered SCFs

**Fig. 4:**
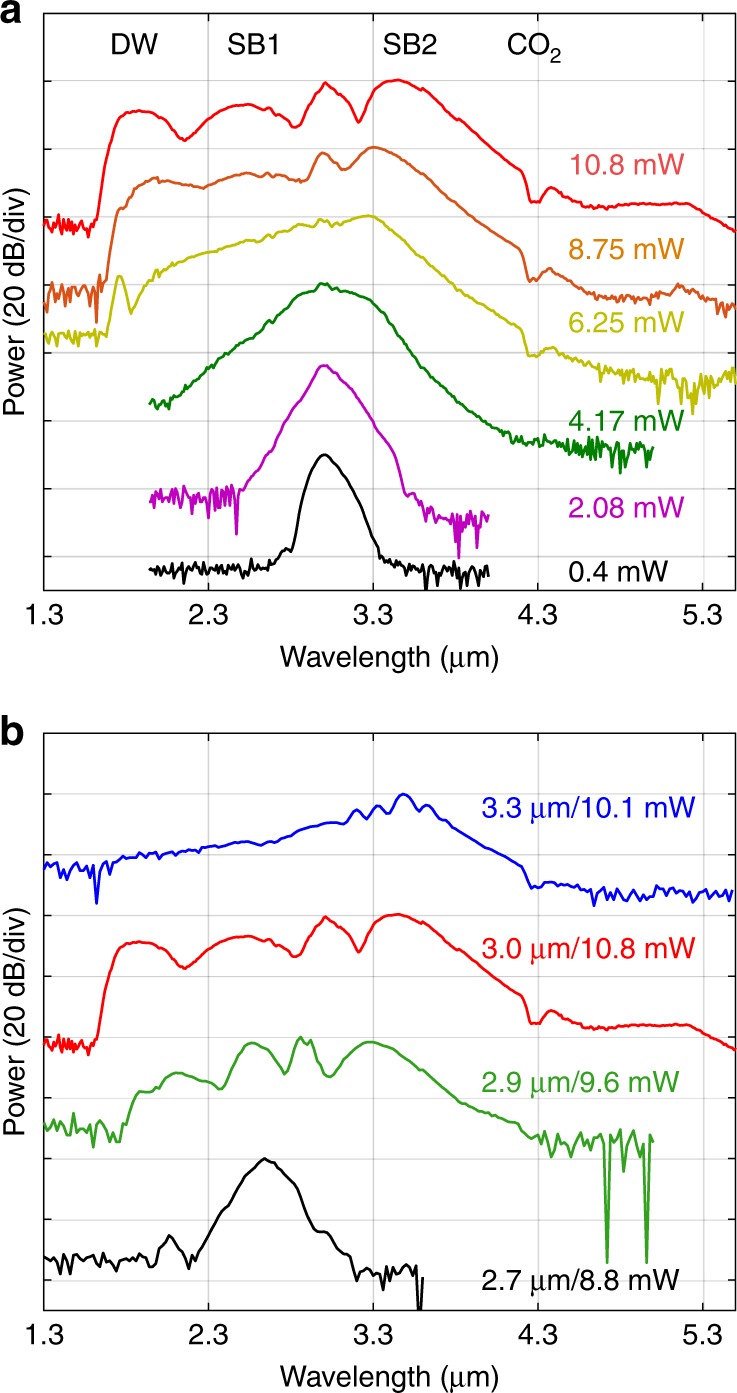
**a** Experimental spectral broadening as a function of coupled average input power. The wavelength converted peaks associated with FWM (sidebands SB1 and SB2) and DW emission are labelled in the top spectrum. The black arrow shows the CO_2_ absorption dip. **b** Comparison of SC generation with different pump wavelengths.

The output SC spectra obtained for coupled average pump powers (see the “Methods” section) increasing from 0.4 to 10.8 mW are shown in Fig. 4a. For the highest power, the spectrum broadened significantly, spanning 1.72 octaves from 1.62 to 5.34 μm at the −40 dB level, for a maximum coupled peak power of only 1.19 kW, as shown in Table 1. We note that owing to multiphoton absorption effects in the silicon core, increasing the power further does not result in a significant improvement to the overall spectral bandwidth. The spectral broadening produced in our SCF is dominated by self-phase modulation (SPM), followed by four wave-mixing (FWM) and dispersive wave (DW) emission, as evident from the evolution of the spectral profile and as labelled in the top spectrum. This evolution is similar to what was previously reported in a deposited silicon fibre^43^ and in SOI nanowires, but the bandwidth is significantly increased (almost twice as broad), and the red spectral edge is well beyond the previous cut-off of 3.3 μm (by ~2000 nm)^12,13^. Moreover, the long wavelength signals (>5.5 µm) in the high power spectrum of Fig. 4a are still above the noise level; however, we were not able to measure beyond this point due to the limited detector sensitivity. The offset of the noise floor at the short and long wavelength edges is due to different noise levels of the two detectors in the monochromator. Owing to the free space coupling arrangement, the atmospheric CO_2_ absorption dip at 4.25 μm can be clearly observed in all spectra when the coupled input average power is above 6 mW. We attribute the extended spectral broadening in our SCF over that in previous reports for planar silicon core/silica clad waveguides, which were limited to an octave span^12,13^, to the low losses at longer wavelengths of the specially designed tapered structure. However, it is worth noting that mid-IR SC spectra with similar bandwidths and longer wavelength edges were obtained in silicon waveguides that are not clad in silica. The first of these was generated in a silicon-on-sapphire waveguide, which benefited from the long wavelength transmission of the sapphire cladding but at the cost of increased fabrication complexity associated with the hardness of this material^16^. More recently, a bulk silicon sample was employed, which negated any issues associated with complex claddings but required a pump power that was three orders of magnitude larger than the typical requirement due to the lack of waveguide confinement^44^.

**Table 1:** Supercontinuum results.

Coupled average power (mW)	Coupled peak power (kW)	Spectral range (μm)	Octaves
10.8	1.19	1.62–5.34	1.72
8.75	0.96	1.66–4.51	1.44
6.25	0.69	1.68–4.45	1.41
4.17	0.46	1.94–3.96	1.03
2.08	0.23	2.48–3.59	0.53
0.41	0.045	2.62–3.37	0.36

*Note*: SC spectral range and octaves are taken with a −40 dB bandwidth

Figure 4b shows the results from tuning the pump wavelength for four different wavelengths near 3 µm. For comparison, all the spectra were recorded at their broadest bandwidth, and the corresponding coupled average pump power is as labelled. As expected, the maximum spectral bandwidth is obtained at the optimum pump wavelength (*λ*_p_ = 3 μm), where the tapered SCF exhibits low anomalous dispersion (*β*_2_ = −0.3 ps^2^ m^−1^). In particular, when pumping in the normal dispersion regime at *λ*_p_ = 2.7 μm, the spectral broadening is mainly induced by SPM, so that the spectrum exhibits the smallest broadening of ∼1100 nm. These results highlight the important role that dispersion plays in enhancing the spectral broadening, with the broadest spectrum being obtained when the phase-matching conditions for DW emission are met. By carefully designing the taper profiles for the desired pump wavelength, the spectral broadening can be tailored for the wavelength region of interest. Nevertheless, all of the top three spectra in Fig. 4b exhibit more than an octave bandwidth at the −40 dB level, which shows that, owing to the range of core diameters accessible in the tapered SCF platform, it is more tolerant to variations in the fabricated dimensions than the straight waveguides where nanoscale precision is required for dispersion engineering^45^.

### Spectral brightness

**Fig. 5:**
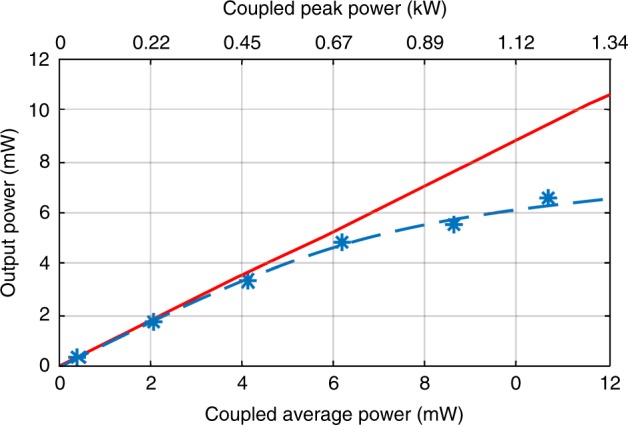
Output SC power versus coupled power, average power (bottom) and peak power (top), for the tapered SCF. The red line indicates the linear loss.

Figure 5 shows the converted SC powers at the output facet of the tapered SCF for increasing coupled average (peak) power up to 10.8 mW (1.19 kW). The converted SC powers are back calculated by using the values measured by the detector and by taking into account the coupling loss at the output facet. A power conversion efficiency (the ratio of the converted SC power *P*_out_ to the coupled average power *P*_in_) as high as ∼61% was achieved when the SC extends over a 3700 nm bandwidth at the −40 dB level. To the best of the authors’ knowledge, this is the highest power conversion efficiency reported in any group IV semiconductor platform^18^ and is competitive even with the non-silica soft glassfibres^28^. The converted SC power reached a value of 6.6 mW for a 10.8 mW coupled average power, resulting in a spectrally bright SC with an average power spectral density of PSD_average_ = 0.002 mW nm^−1^. The simulated SC powers (dashed curve), including multiphoton absorption (see the “Methods” section), show good agreement with the measurements. Unlike previous SC spectra obtained from similar coupled peak intensities (34.9 GW cm^−2^)^16,46^, the total nonlinear loss introduced by the SCF was only 1–2 dB due to the short taper waist length. When combined with the high output coupling efficiency, an SC power of ∼4 mW was obtained outside the fibre. This is a significant improvement over previous mid-IR SC demonstrations in silicon waveguides, which were limited to out-coupled powers <1 mW^16^. Notably, the output power of the SC obtained from our SCF is sufficient for use in state-of-the-art mid-IR gas spectroscopy applications^47^. Moreover, as the pump power is far below the damage threshold of crystalline silicon, stable operation is expected, and the tapered SCF used in this experiment exhibited no measurable change in transmission over several months of experiments.

### Numerical simulations and coherence properties

**Fig. 6:**
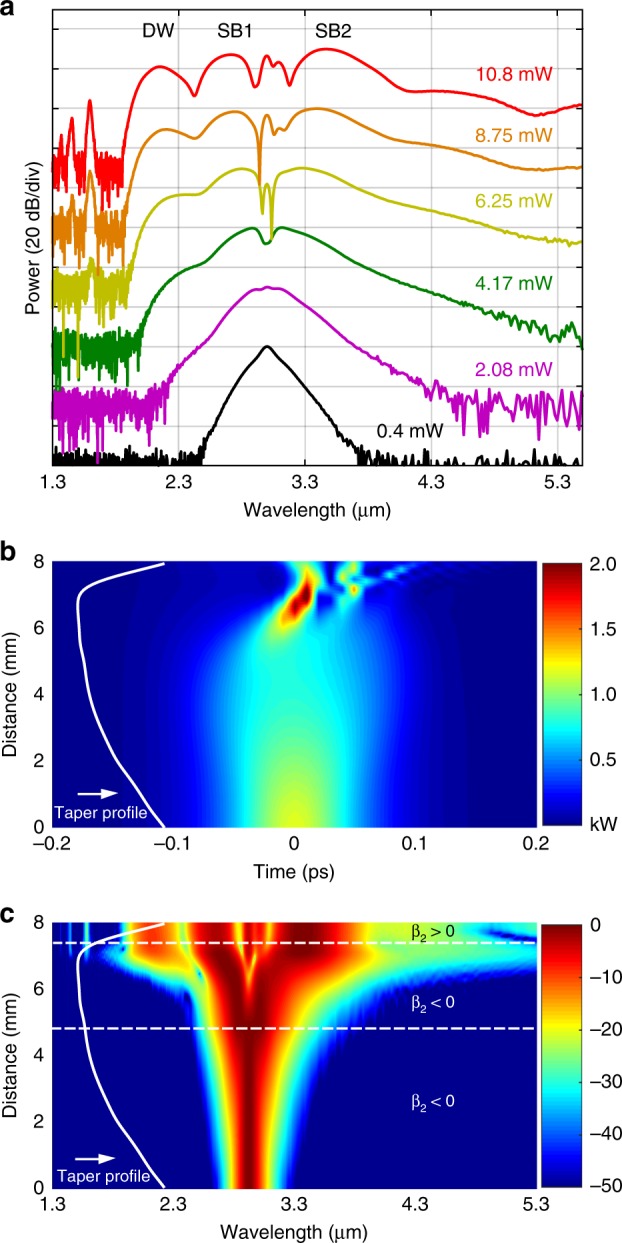
**a** Numerical simulation results for SC spectra generated in a tapered SCF. **b** Simulated temporal evolution of the SC along the tapered fibre with a maximum coupled peak power of 1.19 kW. The colour bar shows the peak power in kW. **c** Simulated spectral evolution of the SC along the tapered fibre on a normalised logarithmic scale.

SC generation and its coherence properties can be simulated by numerically solving a generalised nonlinear Schrödinger equation (NLSE). As a starting point, Fig. 6a shows the simulated spectra as a function of pump power for comparison with the measured spectra in Fig. 4a. The wavelength-dependent linear loss, second-order and third-order dispersions (*β*_2_, *β*_3_), nonlinear refractive index (*n*_2_), 3PA, free-carrier absorption (FCA) and dispersion (FCD) were included in the model (see the “Methods” section). The simulated spectra show very good agreement with the measured results, both in terms of their bandwidths and their spectral features. For example, the −40 dB bandwidth of the simulated SC for 10.8 mW input power is 3600 nm (1.9–5.5 µm), which is almost identical to the experimental result shown in Table 1, and the three main spectral peaks (SB1, SB2 and DW) appear at similar positions. The slight difference between the simulations and experiments arises in the position of the DW, where there is a mismatch of ∼0.2 μm. This discrepancy may be caused by uncertainties in the higher order dispersion values used in the simulations, principally due to the difficulty in precisely mapping the core diameter variations along the taper profile and waist. In addition, the discrete DW peaks on the short-wavelength side are not visible in the experimental spectra because they are relatively weak and far away from the optimised coupling wavelength.

To better understand the role of the tapered profile, Fig. 6b, c show the simulated temporal and spectral evolutions along the SCF for the maximum coupled peak power of 1.19 kW, clearly illustrating the complete spectral dynamics. The SC is generated through three steps. In the first section, between 0 and 4 mm of the fibre, the pump pulse is propagated in the normal dispersion regime and exhibits modest broadening due to SPM. As the core diameter continues to decrease, the pump pulses access the anomalous dispersion region and eventually reach the waist region at 5.5 mm. In this section, efficient phase matching occurs for the FWM process, which leads to pulse compression, break-up and eventually DW emission to produce a broadband SC spectrum. The final section incorporates the sharp, 1 mm long, up-taper to allow the SC light to be collected with minimal loss, during which there are no significant changes to the spectral components. It is worth mentioning that although this taper design was applied to our SCF platform, a similar approach can also be exploited to extend mid-IR SC in planar SOI-based waveguides or any other semiconductor waveguide platforms.

**Fig. 7:**
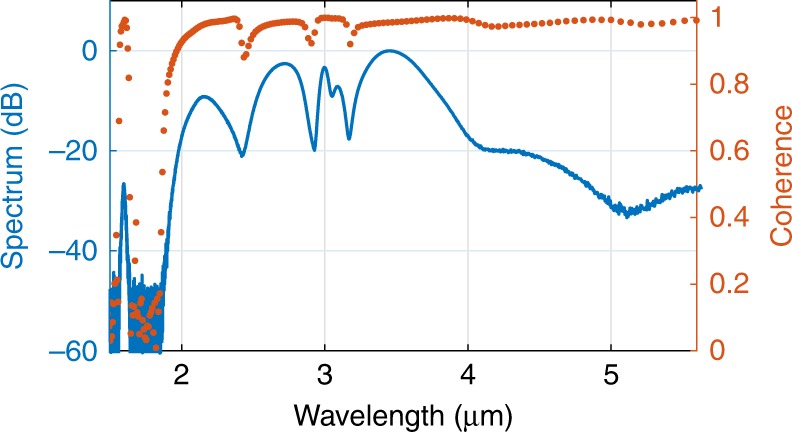
Simulated coherence with quantum noise and ±5% power fluctuations shown in orange, together with the simulated SC spectra (blue) for the highest input power in Fig. 4a.

For applications such as spectroscopy, optical frequency comb metrology, and optical coherence tomography (OCT), it is important to ensure that the generated SC can preserve the coherence of the pump laser. Unlike SC sources that make use of picosecond or longer pump pulses, where the broad spectral bandwidth is generated via amplification of background noise (modulation instability)^48^, by relying largely on SPM and FWM, the SC generated in Fig. 4a is expected to have maintained good coherence, as reported in refs. ^12,17^. The coherence of the SC was simulated via the method described in ref. ^12^ using the mutual and self-coherence functions (see Supplementary Information Section V) by including 200 different SC spectra. Each SC spectrum is generated by incorporating a 5% intensity variation together with quantum noise, which is included as one photon per mode with a random phase, into the input pulse envelope^48^. As shown in Fig. 7, the SC generated in this tapered SCF is highly coherent (>0.9) and close to unity over its entire bandwidth, except for the region containing the low power discrete DWs on the short wavelength side. This is in good agreement with the predictions that SC spectra pumped by femtosecond pulses largely preserve the coherence of the pump source^49^. The coherence can be further improved to approach unity if the SCF is pumped by a more stable laser source (e.g., a mode-locked fibre laser) with negligible power fluctuations and intensity variations^50,51^, which is a further compelling reason to move towards an all-fibre system. Moreover, phase-coherent frequency comb generation can also be achieved based on this octave-spanning mid-IR SC by replacing the pump OPO with a comb seed source^12^. Additional numerical simulations were conducted to verify this (see Supplementary Information Section VI), whereby a clear comb structure can be observed in the SC spectra when pumped with a pulse train constructed using the parameters given in Fig. 6a.

### Selective spectral improvement

**Fig. 8:**
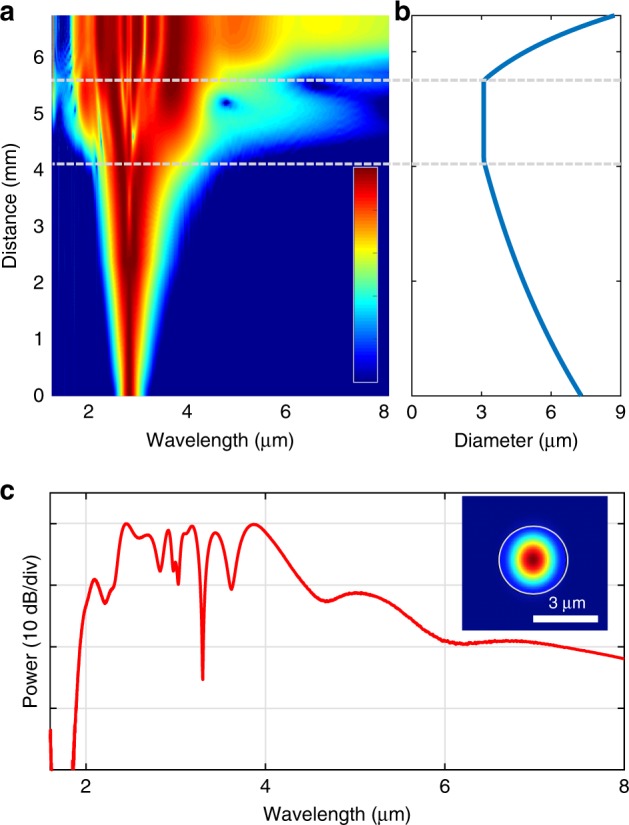
**a** Simulated spectral broadening within a tapered SCF proposed for long wavelength generation. **b** Tapered SCF profile. **c** Simulated SC output spectrum showing more than two octaves of broadening. Inset: mode image in the tapered waist at a wavelength of 8 μm.

The experimental results showed that the tapered SCF platform is capable of generating SC spectra extending over 55% of the transparency window of the silicon core material. To access the remaining wavelength region, further optimisations of the taper designs were numerically investigated. In silicon-based waveguides, two of the most important contributing factors that determine the SC spectral coverage are the nonlinear absorption and the waveguide dispersion profile. As shown in Fig. 5, the nonlinear absorption in the silicon core eventually saturates the output power for increased pump powers, which ultimately limits the bandwidth. Hence, the best route to expand the SC is through new taper designs. To extend the blue edge, the most efficient route is to reduce the taper waist and decrease the pump wavelengths, which shifts the position of the DW to shorter wavelengths. However, this comes at the expense of cutting off the longer wavelength light due to increased interaction with the lossy cladding^52,53^. As the emphasis of our work is on extending the long wavelength edge, a taper was designed with a slightly larger waist diameter (3.1 μm) and a shorter overall length (see Supplementary Information Section VII), as shown in Fig. 8b. Even with the high linear losses of the silica cladding for the longer wavelengths, the simulated spectrum shown in Fig. 8b can still reach beyond 8 μm at approximately the 20 dB level. In this instance, the fibre is pumped with an average input power of 27.2 mW, corresponding to a peak power of 3 kW, which is a more readily accessible power level than the MWs typically used for pumping the high nonlinearity glass fibres^6^, owing to the tight mode confinement of our SCF. However, the modelling is limited to wavelengths up to 8 μm, as the absorption in the silicon core increases substantially beyond this value. As shown in Fig. 8a, the longer wavelength components (beyond 6 μm) are generated in the up-tapered output region. Although this is beneficial for minimising the long wavelength interaction with the cladding, it means that the broadening is very sensitive to variations in the output taper diameter, as higher order dispersion plays a key role in the frequency conversion. Unfortunately, reproducing these results experimentally is currently restricted by the performance of the tapering instrument, which cannot accurately produce such an SCF design. Nevertheless, this simulation indicates that the tapered SCF platform has the potential to produce SC spectra that cover the entire silicon transparency window, even with absorptive silica cladding. Further work may also consider replacing the core and cladding materials in a similar way to what is being explored in planar platforms^16,18^, though at the cost of introducing more fabrication complexity.

## Discussion

A compact SCF platform was demonstrated that achieves low loss transmission across the mid-IR spectral regime. By exploiting a novel asymmetric taper design, a coherent SC span of 1.74 octaves was generated from 1.6 to 5.3 μm, which is the broadest SC reported in a silicon core/silica-clad waveguide. The generated mid-IR spectrum covers the first infrared atmospheric window of 3–5 μm, which includes absorption bands for many greenhouse gases, such as carbon monoxide (CO), nitrous oxide (N_2_O), and carbon dioxide (CO_2_)^47^. Thus, such a broadband SC with high brightness and coherence can be utilised for practical mid-IR applications in the areas of spectroscopy and free-space communications^54^. The experimental results, together with numerical simulations of an optimised profile, show that this tapered SCF platform can be used to generate SC spectra that cover the entire silicon transparency window up to 8 μm. Continued efforts to reduce loss and optimise the integration of this platform with other mid-IR fibre components allows for the construction of robust, high power, and practical all-fibre SC-based mid-IR sources.

## Methods

### Fibre fabrication and post processing

The SCFs used in the experiments were fabricated using the MCM, whereby a silicon rod was enveloped inside a silica glass capillary to form a preform, which was subsequently drawn down into a fibre. The silica capillary was coated with a thin layer of calcium oxide (CaO), which forms an interface between the core and cladding during the drawing process. This layer plays an important role, as it prevents the dissolution of silica from the cladding into the silicon core and reduces the thermal strain arising from high-temperature processing. The core material used in this work was a high-resistance silicon rod (slightly P-doped, *R* > 4800 Ω cm) with negligible free carrier density. The fibre was drawn at a temperature of ~1950 °C with a drawing speed of ~25 m min^−1^. The as-drawn silicon core materials are polycrystalline in nature, with crystalline grain sizes of a few hundreds of micrometres to millimetres in length. The as-drawn fibres have a silicon core diameter of ∼20 µm with a ~140 µm silica cladding. The optical transmission losses of these fibres are ~10 dB cm^−1^ within the telecom band.

To improve the optical properties, the fibres can be thermally annealed or tapered using standard glass processing systems. Here, a Vytran GPX3400 was used to taper the fibres, as this allows for control of the core dimensions post-draw. As the silicon core is completely molten during the tapering process, it is possible to use the drawing speed and temperature to control the cooling dynamics, which promotes large grain (centimetre length) crystal growth in the core^31^. The core/cladding interface of these fibres is also extremely smooth (root-mean-square roughness of ∼0.7 nm), so small core fibres (diameters < 1 μm) can be obtained with minimal scattering and absorption losses^31^.

### Optical characterisation

Due to the variation in the core size along the length, the linear optical transmission losses of the tapered SCF were characterised by a single pass measurement. The fibre was mounted in a capillary tube and polished by using routine fibre preparation methods. A femtosecond OPO was employed as the light source to cover the full wavelength range of 1.7–3.7 µm. Thus, to ensure that nonlinear absorption was avoided, the average power of the injected light was kept below 100 µW. The light was launched into the core of the tapered SCF using the same configuration as that in the SC measurements. The output of the fibre was imaged using a mid-IR camera to confirm that transmission occurred only through the silicon core. The coupling was optimised using a set of Thorlabs Nanomax stages. The power of the input and output beams was measured using two detectors: an InGaAs photodiode power sensor (Thorlabs S148C) for wavelengths <2.5 µm and a high-resolution optical thermal power sensor (Thorlabs S320C) for wavelengths above 2.5 µm. The coupled average powers used in this work are the those inside of the fibre. These values can be obtained from the powers measured at the input of the waveguide by taking into account the coupling efficiency (including the losses from mode mismatch, reflection and lens). The coupled peak powers can then be calculated using the pulse width (*T*_FWHM_) and repetition rate (PRR) of the pump source as

$${P}_{{\mathrm{peak}}} = \frac{{{P}_{{\mathrm{avg}}}}}{{{T}_{{\mathrm{FWHM}}} \cdot {\mathrm{PRR}}}}$$

### Simulations

The nonlinear Schrodinger equation (NLSE) was used to model the pulse propagation along the tapered SCFs. A simplified model was employed, which includes the effects of 3PA, but not TPA, owing to the spectral position of the pump wavelength^16^:

$$\frac{{\partial {A}}}{{\partial {z}}} + \frac{{{\mathrm{i}}\beta _2\partial ^2{A}}}{{2\partial {t}^2}} - \frac{{{\beta }}_3\partial ^3{A}}{{6\partial {t}^3}} = {\mathrm{i}}k_0{n}_2\frac{{|{A}|^2}}{{{A}_{{\mathrm{eff}}}}}{A} - \frac{{{\beta }}{_{3{\mathrm{PA}}}|{A}|^4}}{{2{A}_{{\mathrm{eff}}}^2}}{A} - \frac{{\sigma }}{2}\left( {1 + {\mathrm{i}}\mu } \right){N}_{\mathrm{c}}{A} - \frac{{\alpha _{\mathrm{l}}}}{2}{A}$$

with

$$\frac{{\partial {N}_{\mathrm{c}}}}{{\partial {t}}} = \frac{{\beta _{3{\mathrm{PA}}}}}{{3hv}}\frac{{|{A}|^6}}{{{A}_{{\mathrm{eff}}}^3}} - \frac{{{N}_{\mathrm{c}}}}{{{\uptau }}_{\mathrm{c}}}$$

Here, *A* is the amplitude of the slow varying envelope of the optical pulse, *z* is the propagation distance, *β*_2_ and *β*_3_ are the second-order and third-order dispersion of the fibre, respectively, *k*_0_ is the propagation constant, *n*_2_ is the nonlinear Kerr coefficient, and *A*_eff_ is the effective mode area. In addition, *α*_l_ is the linear wavelength-dependent loss; *β*_3PA_ is the 3PA coefficient, and *σ*, *μ*, *N*_c_, and *τ*_c_ are the FCA, FCD, and 3PA-induced free carrier densities, and the free carrier lifetime. The values of these parameters can be found in Supplementary Information Section IV. Here, *h* is Planck’s constant and *v* is the centre frequency of the pulse. This model only includes the second-order and third-order dispersion terms, since higher orders of dispersion typically contain large errors due to uncertainties in the wavelength-dependent refractive index of the polycrystalline material and the precise core diameters. The Raman effect is not included in our simulations, as it has a negligible impact on the generated SC and its cohenrence^48^. The equation was solved numerically using the well-known split-step Fourier method.

## Supplementary information

Supplementary Information for Low-loss silicon core fibre platform for mid-infrared nonlinear photonics
