# Supplementary material for: Low-loss silicon core fibre platform for mid-infrared nonlinear photonics
Source: Light Sci Appl. 2019 Nov 21;8:105. doi: 10.1038/s41377-019-0217-z (PMC6872570; doi:10.1038/s41377-019-0217-z)
Supplement: Supplementary file 1 — Supplementary Information for Low-loss silicon core fibre platform for mid-infrared nonlinear photonics [file 41377_2019_217_MOESM1_ESM.pdf]

Supplementary Information for

Low-loss silicon core fibre platform for mid-infrared nonlinear photonics

Haonan Ren[1], Li Shen[1, 2, *], Antoine F. J. Runge[1, 3], Thomas W. Hawkins[4], John Ballato[4], Ursula Gibson[5, 6] and Anna C. Peacock[1]

[1]Optoelectronics Research Centre, University of Southampton, Southampton, SO17 1BJ, UK

[2]Wuhan National Laboratory for Optoelectronics (WNLO), Huazhong University of Science and Technology, Wuhan 430074, China

[3]Currently with the Institute of Photonics and Optical Science (IPOS), School of Physics, University of Sydney, NSW 2006, Australia

[4]Department of Materials Science and Engineering, Clemson University, Clemson, South Carolina 29634, USA

[5]Department of Physics, Norwegian University of Science and Technology (NTNU), N-7491 Trondheim, Norway

[6]Department of Applied Physics, KTH Royal Institute of Technology, Stockholm 10044, Sweden

*Corresponding author: Lishen@hust.edu.cn Tel: +44 (0)23 80593155 Fax: +44 (0)23 80593142

I. Design of tapered silicon core fibre (SCF) for mid-infrared supercontinuum generation (SCG)

Key to achieving a broad spectral coverage in our tapered SCFs is to balance the nonlinear pulse dynamics with the overall losses of the fibre (both linear and nonlinear). This has led us to establish the following design criteria:

1. Large input and output cores to facilitate light coupling.
2. An input down-taper transition that is long enough to induce some initial spectral broadening, which helps to seed further nonlinear processes.
3. A tapered waist with a diameter that is close to the zero dispersion of the pump and a length that is long enough to induce a large nonlinear phase shift.
4. An up-taper transition at the output that is as short as possible to minimize absorption of the longer mid-infrared wavelengths.

For a taper with three main sections, input taper, taper waist, and output taper, there are six key parameters to optimize. These are diameters of the input facet (D_{in}), waist (D_w) and output facet (D_{out}), as well as the lengths of the input transition region (L_{Td}), the waist (L_{waist}) and the output taper transition (L_{Tu}), as shown in Fig. S1a. As these parameters are connected through the complex nonlinear pulse evolution in the fibre, we have used extensive modelling of the NLSE to establish the design that produces the broadest supercontinuum. To simplify the problem, modelling of each section is initially conducted independently in three steps, and then combined at the final stage to verify the overall performance.

Firstly, we choose the waist diameter and length. The waist diameter is chosen such that it is close to the zero-dispersion wavelength (ZDW), in the anomalous dispersion regime, and yet still has relatively low losses (<3 dB mm^{-1}) for wavelengths up to 7 µm. For a pump at 3 µm, the waist diameter should be in the range ~2.5-3 µm (see Fig. 1b and 2b). The waist length is then chosen from the results of NLSE modelling, as shown in Fig. S1b. From these results, we target a length of 2 mm as this corresponds to maximum broadening but is also before the total transmission losses become too large.

The second step is to design the input down-taper transition. The input diameter is set to be 10 µm to allow for efficient coupling, thus we only need to choose the length. Fig. S1c shows three taper profiles with lengths 0, 2.5, and 5 mm, with their corresponding spectra in Fig. S1d. It is clear from this that the longest taper 5 mm produces the broadest spectrum with the longest red spectral edge. We note that longer lengths do not offer any additional broadening as the losses also increase.

The final step is to select the dimensions for the up-taper output transition region. As for the input, the output facet diameter is set to 10 µm, but now the length is chosen to be as short as we can fabricate to minimize the losses of the long wavelength light generated in our continuum.

Using these criteria, we conducted NLSE modelling of the full taper profile to confirm that these criteria led to the broadest SC with the longest red wavelength edge. Several tapered SCFs were then fabricated, from which we selected the taper that was closest to our design. This resulted in a fibre with the dimensions as follows:

Input taper: input diameter D_{in} =10 µm, length L_{Td} = 5.5 mm

Waist: diameter D_w = 2.8 µm, length L_{waist} = 1.7 mm

Output taper: output diameter D_{out} = 10 µm, length L_{Tu} = 1 mm.

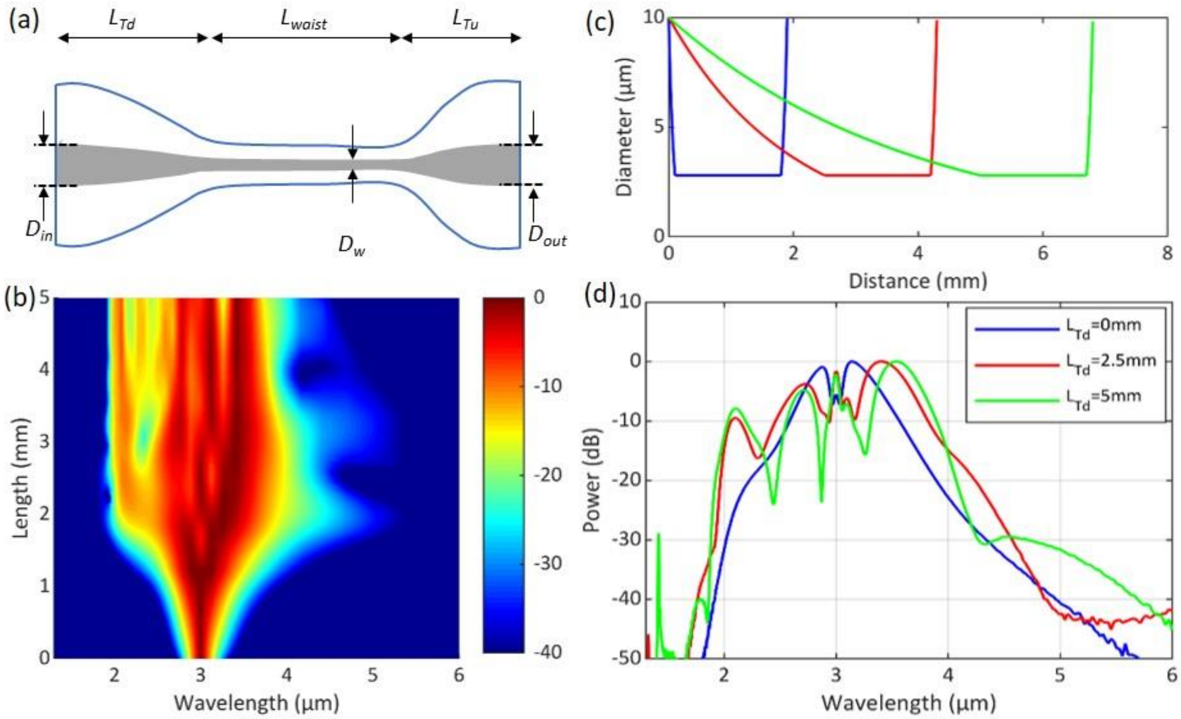

Figure S1 (a) Schematic of the asymmetric taper design. (b) Simulation of SCG evolution in a straight SCF with a 2.8 µm core diameter and 1500 W coupled pump power at 3 µm. (c) Taper profile with different down transition lengths. (d) Simulated SCG spectra corresponding to the profiles in (a).

II. Transmission of the device

To characterise the transmission properties of the tapered SCF, the insertion loss of the device was measured for OPO wavelengths between 1.7-3.7 µm, using the lowest power setting to minimise the effect of nonlinear absorption, as shown in Fig S2. The transmission of the fibre was then estimated by excluding the 1.5 dB reflection from the facets and 2 dB losses induced from the two coupling lenses (L1 and L2). The large error bars are due to inconsistent coupling losses that vary for the different input wavelengths.

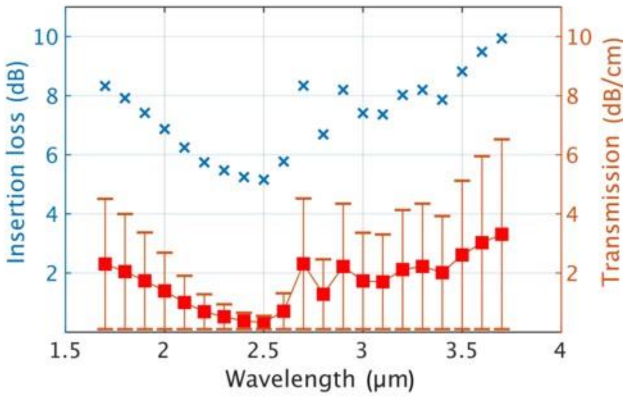

Figure S2 Transmission properties of the tapered SCF at different wavelength.

III. Mode launching conditions

In the experiments, the pump light was free-space coupled into the fibre using an objective lens, which was chosen to produce a beam waist that best matched the fundamental mode radius. The focused beam size (assuming a Gaussian profile) and its overlap with the guided modes of the SCF are calculated using commercial software (Lumerical mode solution) for the pump wavelength of 3 µm. As shown in Fig. S3, when the focused beam radius is 2.7 - 5 µm, most of the power is coupled into the LP_{01} and LP_{02} modes, with more than 95% in the LP_{01} mode alone. Thus by choosing the appropriate lens to obtain a focused beam radius of ~3 µm, we would expect strong coupling to the fundamental mode.

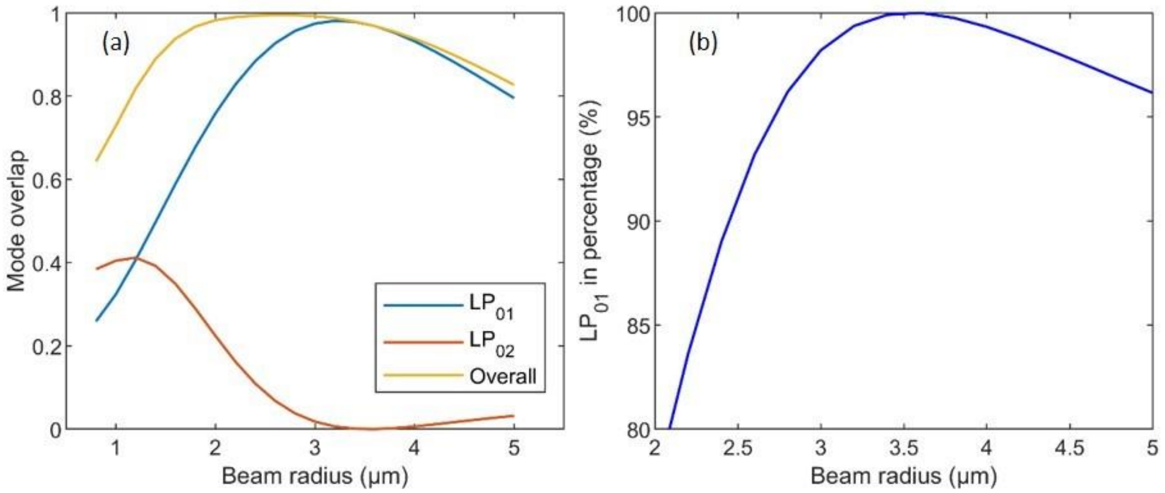

Figure S3 (a) Mode overlap of a Gaussian input beam and the two lowest order LP modes in the SCF with 10 µm core diameter at a 3 µm pump wavelength. (b) Percentage of the overall coupled power in the fundamental mode for different input beam waist radii.

Confirmation that the beam profile from the OPO is highly Gaussian is provided in Fig. S4a. At the pump wavelength of 3 µm, the beam diameter is ~ 3 mm, corresponding to a ~ 2.9 µm focused beam waist radius at the fibre facet using L1 (NA = 0.56). From Fig. S3b, we expect that for this configuration we have at least 95% power coupled into the fundamental mode. The output beam profile of the near infrared component of the generated supercontinuum was imaged using a CCD camera (MicronViewer 7290A up to 2.2 µm), as shown in Fig. S4b. The measured output beam has retained its Gaussian shape, even at the shorter wavelengths, which further confirms that the light was primarily coupled into fundamental mode of our tapered SCF.

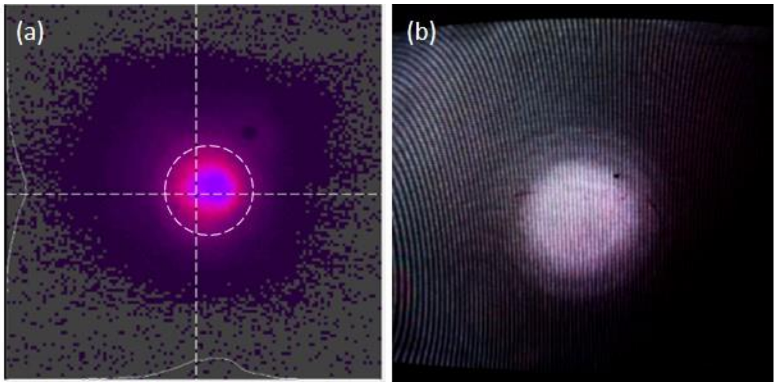

Figure S4 (a) Measured input beam profile, the dashed lines are guides to eyes. The lines on the boundary of the image show the beam profiles in the x (horizontal) and y (vertical) directions. (b) CCD image of the output beam profile of the near infrared component.

IV. Simulation parameters

The optical parameters used in the numerical simulations are shown in Table S1. The wavelength dependent loss was used in the simulation from the Fig. S2. For input pulses with a duration of 100 fs, FCA and FCD were found to play a negligible role to the pulse shaping. The simulation uses z dependent dispersion and effective mode area profiles, as shown in Fig. S5a and Fig. S5b, respectively.

Table S1. SC simulation parameters

Parameter	Symbol	Value (unit)
Central wavelength	λ	3 μm
Linear loss	α_l	Wavelength dependent losses shown in Fig. S2
Kerr effect coefficient	n_2	3.0×10^{-18} m^2W^{-1} from Ref. 1
Pulse width	T_{FWHM}	100 fs (hyperbolic secant)
Carrier life time	τ	10 ns
FCA parameter	σ	2.9×10^{-21} m^2 from Ref. 2
3PA coefficient	β_{3PA}	2×10^{-27} m^3W^2 from Ref. 1,3
GVD parameter	β_2	Shown in Fig. S5a
3rd order dispersion	β_3	Shown in Fig. S5a
Effective mode area	A_{eff}	Shown in Fig. S5b

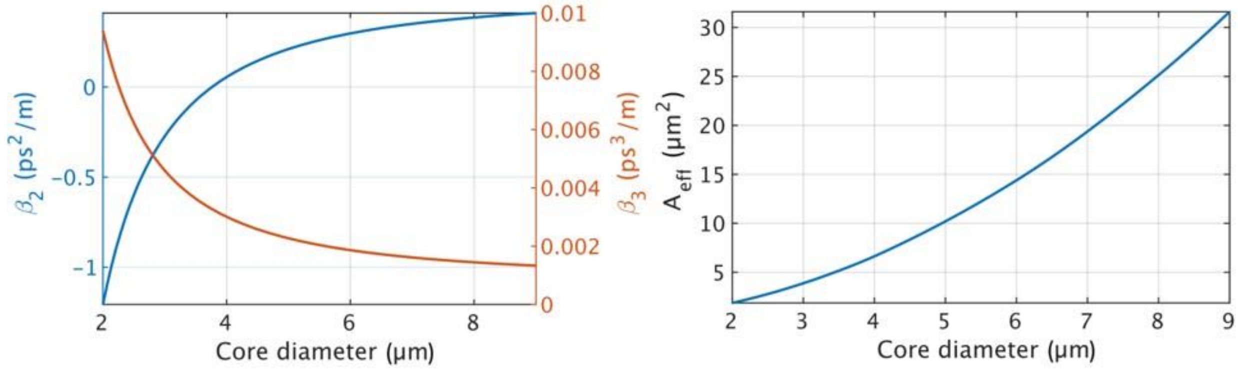

Figure S5 (a) Dispersion properties of the tapered SCF at the pump wavelength of 3 μm. (b) Effective mode area of the tapered SCF at the pump wavelength of 3 μm.

V. Coherence calculation

Coherence of the SC is calculated by the equation in Ref. 4:

$$|g_{12}(\lambda)| = \left| \frac{\langle A_1^*(\lambda) A_2(\lambda) \rangle}{\sqrt{\langle |A_1(\lambda)|^2 \rangle \langle |A_2(\lambda)|^2 \rangle}} \right|,$$

where A_1 and A_2 are the electric field amplitudes of two independent SC output. g_{12} is the complex degree of first-order coherence and the angle bracket represents the ensemble average over all independently generated SC pairs. In this work, the coherence is calculated by applying 200 individual SC output with this equation. The laser source (OPO) used in this work has a 5% intensity noise with 30 dB level signal to noise ratio. To model the quantum noise, each of spectra was generated by incorporating one photon per mode with random phase on the input pulse envelop.

VI. The simulation of the frequency comb generation

To investigate the suitability of our SCF to be used for mid-infrared comb generation, numerical simulations were conducted using the generalized NLSE (see methods) with a pulse train for the input. The pulse parameters (duration and power) were chosen to be the same as our experimental parameters (10.8 mW input in Fig. 6a), including shot to shot noise, and the

constructed train consisted of 10000 pulses at a repetition rate of 80 MHz. In order to simulate the frequency comb, the SC spectrum was calculated with a 80 kHz resolution (10 kHz for inset) using a simplified algorithm in Ref.5. The comb structure of the supercontinuum spectrum can be seen in Fig. S6a. Close-up views of two selected spectral regions located at A (142.8 THz/2.1 µm) and B (66.6 THz/4.5 µm) are shown with a sampling bandwidth of 400 kHz in Fig. S6b and c, respectively. The generated SC comb spectrum in the SCF clearly spans more than an octave in frequency, owing to the coherence preserving nature of our nonlinear process. The extinction ratios of the comb lines are found to be greater than 30 dB, which is suitable for high-precision spectroscopy applications.

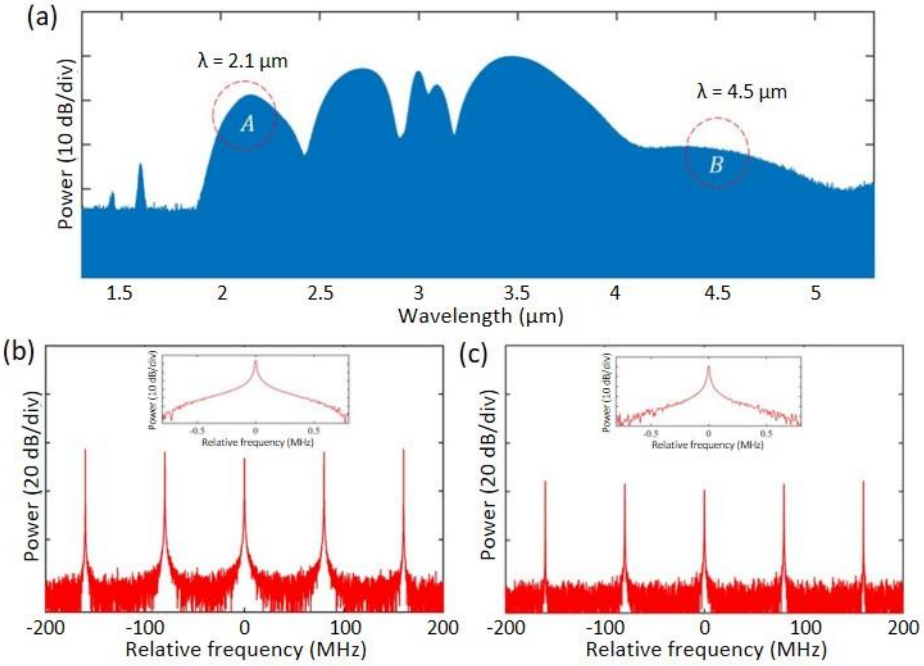

Figure S6 (a) SC-based frequency comb obtained with 10000 input pulses with a repetition rate of 80 MHz. Close-up views of spectra simulated in the vicinity of (b) 142.8 THz/2.1 µm and (c) 66.6 THz/4.5 µm, with sampling bandwidths of 400 kHz. The insets show a single comb line with a 1.6 MHz sampling bandwidth and 8 kHz resolution.

VII. Taper design for long wavelength

In order to generate longer wavelengths up to 8 µm for the same λ= 3 µm pump source, we apply a similar design process to what has been described in Section I. The main features of the new design are that the taper waist diameter is increased slightly (3.1 µm) and the overall fibre length shorten to 6.7 mm, which helps to minimize interaction of the mid-IR light with the cladding to reduce the transmission losses. More specifically, the fibre is gradually tapered down from a 7.3 µm core over the first 4.1 mm to reach the diameter waist, of 1.4 mm length, followed by a 1.2 mm long inverse taper back up to a 9.3 µm core at the output. For an average input power of 27.2 mW (peak power of 3 kW), the generated SC could extend up to 8 µm at around the 20 dB level, as shown in the top spectrum of Fig. S6b. From the temporal dynamics in Fig. S6a, it is seen that the pulse compresses in the first 4 millimeters before breaking up in the waist region. Subsequently, the change of dispersion (anomolous to normal) in the up transition section of the taper enhances the pulse interactions, which further broadens the spectra. In this case, the dispersion profile needs to be precisely tailored to efficiently control the pulse interactions. Hence, this design is very sensitive to the ouput taper profile and has not yet been realised using our existing tapering equipment. Fig. S6b shows the spectrum over different coupled peak powers.

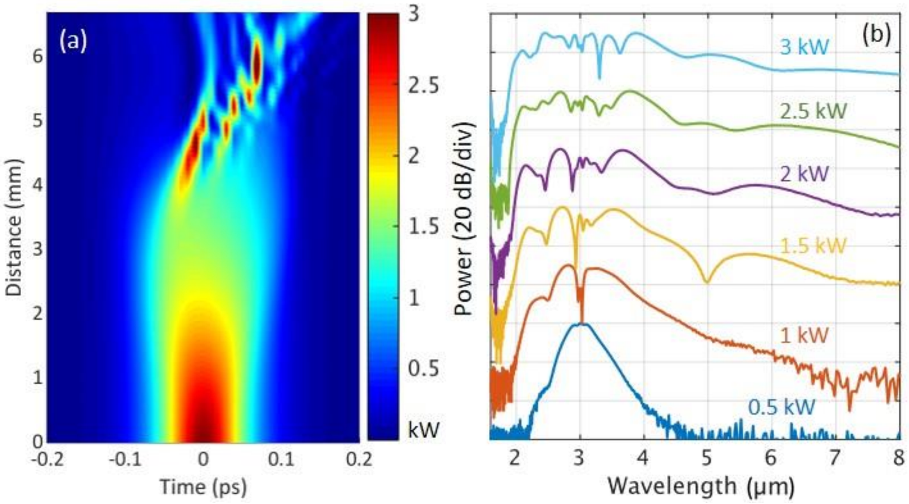

Figure S7: Simulation results for the optimised taper structure. (a) Pulse evolution along the SCF in the time domain. (b) Simulated SC spectra with different coupled peak powers.

Supplementary references:

1. Wang, T. *et al.* Multi-photon absorption and third-order nonlinearity in silicon at mid-infrared wavelengths. *Optics Express* **21**, 32192-32198 (2013).
2. Degallaix, J. *et al.* Bulk optical absorption of high resistivity silicon at 1550 nm. *Optics Letters* **38**, 2047-2049 (2013).
3. Pearl, S. *et al.* Three photon absorption in silicon for 2300-3300 nm. *Applied Physics Letters* **93**, 131102 (2008).
4. Dudley, J. M. *et al.* Coherence properties of supercontinuum spectra generated in photonic crystal and tapered optical fibers. *Optics Letters* **27**, 1180-1182 (2002).
5. Kuyken, B. *et al.* An octave-spanning mid-infrared frequency comb generated in a silicon nanophotonic wire waveguide. *Nature Communications* **6**, 6310 (2015).
